# Direct design of ground-state probabilistic logic using many-body interactions for probabilistic computing

**DOI:** 10.1038/s41598-024-65676-z

**Published:** 2024-07-02

**Authors:** Yihan He, Sheng Luo, Chao Fang, Gengchiau Liang

**Affiliations:** 1 Department of Electrical and Computer Engineering, National University of Singapore, Singapore, 117576 Singapore; 2 Industry Academia Innovation School, National Yang-Ming Chiao Tung University, Hsinchu City, 300093 Taiwan

**Keywords:** Probabilistic computing, Ground-state probabilistic logic, Binary energy landscape, Many-body interactions, Invertible multiplier, Electrical and electronic engineering, Applied physics, Statistical physics, thermodynamics and nonlinear dynamics

## Abstract

In this work, an innovative design model aimed at enhancing the efficacy of ground-state probabilistic logic with a binary energy landscape (GSPL-BEL) is presented. This model enables the direct conversion of conventional CMOS-based logic circuits into corresponding probabilistic graphical representations based on a given truth table. Compared to the conventional approach of solving the configuration of Ising model-basic probabilistic gates through linear programming, our model directly provides configuration parameters with embedded many-body interactions. For larger-scale probabilistic logic circuits, the GSPL-BEL model can fully utilize the dimensions of many-body interactions, achieving minimal node overhead while ensuring the simplest binary energy landscape and circumventing additional logic synthesis steps. To validate its effectiveness, hardware implementations of probabilistic logic gates were conducted. Probabilistic bits were introduced as Ising cells, and cascaded conventional XNOR gates along with passive resistor networks were precisely designed to realize many-body interactions. HSPICE circuit simulation results demonstrate that the probabilistic logic circuits designed based on this model can successfully operate in free, forward, and reverse modes, exhibiting the simplest binary probability distributions. For a 2-bit × 2-bit integer factorizer involving many-body interactions, compared to the logic synthesis approach, the GSPL-BEL model significantly reduces the number of consumed nodes, the solution space (in the free-run mode), and the number of energy levels from 12, 4096, and 9–8, 256, and 2, respectively. Our findings demonstrate the significant potential of the GSPL-BEL model in optimizing the structure and performance of probabilistic logic circuits, offering a new robust tool for the design and implementation of future probabilistic computing systems.

## Introduction

Probabilistic computing^1–4^, an emerging interdisciplinary field, integrates cutting-edge research findings from various domains, including probability theory, statistics, and computer science. The advent of tunably stochastic nanodevices, colloquially known as probabilistic bits (p-bits), has injected new vitality into the development of this field. These p-bit devices, represented by stochastic magnetic tunnel junctions^5–11^, are characterized by their intrinsic stochasticity and the capability for rapid fluctuations on sub-nanosecond timescales^12,13^. These unique properties make p-bits ideally suited to serve as the fundamental building blocks for probabilistic logic networks, enabling the efficient exploration of vast solution spaces and the solving of hard computational problems. By leveraging energy-based computational models^14,15^, researchers have successfully constructed various probabilistic logic circuits. For example, the development of solvers for integer factorization^5,6,16–21^ and Boolean satisfiability^16,22–26^ highlight the immense potential of probabilistic logic in the field of optimization and decision-making.

The core principle underpinning probabilistic logic networks lies in the concept of ground-state computation; hence, they can also be referred to as ground-state probabilistic logic (GSPL). In physics, the energy landscape serves as a conceptual framework that delineates the potential energy levels within a system, with the state possessing the lowest energy being identified as the ground state. Analogously, in the context of GSPL, for small-scale target logic, the Boolean function can be mapped onto the energy landscape using energy-based models such as the Ising model^14^ or fully connected Boltzmann machines^15^, based on its known truth table. For larger-scale GSPL circuits, they can be constructed by combining these pre-mapped fundamental gates. When a GSPL network circuit reaches thermal equilibrium, the probabilities of all potential solutions converge according to their respective energy levels, following the Boltzmann distribution. Consequently, by simply identifying the state configuration with a relatively high probability, the solution to the problem can be determined.

However, the current design framework for GSPL still faces several pressing issues. Firstly, most existing works, when using energy-based models for mapping, only consider pairwise interactions between p-bits^6,20,21,27^, which limits the descriptive capability of the energy-based model for the energy landscape of the target GSPL gates. This leads to two unfavorable consequences: (1) For logic gates that can be directly mapped to corresponding GSPL, although the correct state configuration that aligns with the truth table can be mapped to the ground state, wrong state configurations will correspond to multiple energy levels of the system, resulting in a complex energy landscape. (2) For logic gates that cannot be directly mapped, such as multi-input logic gates, additional auxiliary nodes need to be introduced to provide more degrees of freedom for describing the energy landscape. These auxiliary nodes can increase the expressive power of the energy model by introducing additional interaction terms, thereby enabling a more accurate description of the energy landscape for complex logic gates. However, this not only introduces more node overhead and dramatically increases the solution space but also further complicates the energy landscape.

To address these challenges, Ising machines and Boltzmann machines involving higher-order interactions^19,24,28–31^ have been explored. In the context of GSPL, linear programming^19,29,32^ has been employed to find the configuration parameters of small-scale GSPL gates involving many-body interactions among p-bits, albeit at the cost of additional algorithmic complexity. Furthermore, for more complex logic circuits, logic decomposition is typically required, followed by logic synthesis^21,32^ based on fundamental GSPL gates. This process introduces additional software overhead, and more critically, during logic synthesis, the energy levels of the fundamental building blocks undergo stacking. Energy level stacking may introduce additional local minima, making the system more prone to suboptimal solutions, thereby reducing computational accuracy. Consequently, it becomes inevitable to employ annealing techniques^16^ such as simulated annealing and parallel tempering to increase the probability of converging to the global optimum. Furthermore, recent years have witnessed the development of various bio-inspired metaheuristic optimization algorithms, like the coronavirus mask protection algorithm^33^, the alpine skiing optimization algorithm^34,35^, the adaptive dragonfly algorithm^36,37^, the grey wolf optimizer algorithm^38^, etc. These advanced metaheuristic algorithms have shown promising results in tackling real-world complex engineering optimization problems, but their potential to improve the computational accuracy and efficiency of GSPL-based circuits remains to be explored in future research.

In this paper, we propose a novel computational model that enables the direct implementation of GSPL incorporating many-body interactions to address the above challenges. This model, termed ground-state probabilistic logic with a binary energy landscape (GSPL-BEL), leverages many-body interactions for logic implementation, effectively reducing the number of p-bits, significantly shrinking the size of the solution space and greatly simplifying the complexity of the energy landscape. Moreover, this model encompasses a comprehensive methodology for converting arbitrary traditional deterministic logic into GSPL-BEL represented by graphic models. This conversion framework has been encapsulated into an executable program, providing a user-friendly tool for seamless integration of GSPL-BEL into Ising model-based networks. Furthermore, to validate the functionality of the proposed model, we conducted extensive circuit simulations employing p-bit devices to emulate the Ising cells and conventional XNOR gates to implement many-body interactions. This advancement opens new possibilities for harnessing the benefits of many-body interactions in logic design and operations, further exploring the potential of the GSPL-BEL model in diverse probabilistic computing applications.

## Ground-state probabilistic logic with a binary energy landscape

### Computational model based on many-body interactions

**Figure 1:**
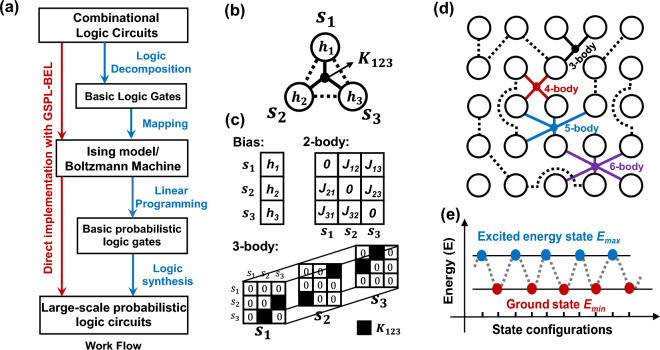
(**a**) Workflow of the proposed GSPL-BEL model (red arrows) and conventional methodology (blue arrows) in designing probabilistic logic circuits. (**b**) Graphical representation and (**c**) mathematical representations of a three-body-interacting GSPL system, which is characterized by the bias term, 2-body and 3-body interaction terms. (**d**) Illustrative graphical model of a more comprehensive GSPL system involving up to 6-body interaction dimensions. (**e**) Schematic diagram of the GSPL’s binarized energy landscape based on the GSPL-BEL model.

The conventional design methodology for combinatorial probabilistic circuits typically encompasses several crucial steps, as illustrated by the blue arrow in Fig. 1a. These steps include logic decomposition, mapping, linear programming, and logic synthesis. Logic decomposition involves breaking down the desired probabilistic circuit into smaller, more manageable components. The mapping process then translates these components into a form compatible with the network’s architecture of the Ising machine or Boltzmann machine. Linear programming is employed to obtain the exact parameters of the network’s configuration. Finally, logic synthesis combines these well-designed components to generate the final probabilistic circuit. In contrast, the GSPL-BEL model offers a more streamlined approach. As depicted by the red arrow, it enables direct mapping from the truth table of the desirable logic circuits to the corresponding GSPL circuits using the Ising model. This direct implementation eliminates the need for the intermediate steps required in the conventional design process.

Figure 1b shows an illustrative graphical model of a three-body interacting system based on the idea of GSPL-BEL. In this system, each node’s state, denoted as *s*, is restricted to binary values of 0 and 1. The GSPL-BEL model distinguishes itself from the conventional Ising model structure, which is limited to pairwise interactions, by incorporating a single branch of three-body interaction. This many-body interaction can be mathematically represented as a tensor *K*_123_, where *K*_123_ = (*s*_1_, *s*_2_, *s*_3_) = (*s*_1_, *s*_3_, *s*_2_) = (*s*_2_, *s*_1_, *s*_3_) = (*s*_2_, *s*_3_, *s*_1_) = (*s*_3_, *s*_1_, *s*_2_) = (*s*_3_, *s*_2_, *s*_1_), as illustrated in Fig. 1c. Figure 1d presents a more comprehensive graphical model derived from the GSPL-BEL. In this system, the maximum dimension of many-body interactions that can be exploited is consistent with the total number of nodes. This design choice allows the dimension of interactions to scale with the number of nodes and the full parameters in designing the system’s network configuration can be captured.

To generalize the GSPL-BEL model, we introduce two vectors: an *M*-dimensional vector, denoted as ***x***, with components *x*_*1*_, *x*_*2*_, … *x*_*m*_, and an *N*-dimensional vector, denoted as ***y***, with components *y*_*1*_, *y*_*2*_, …, *y*_*n*_. These vectors represent the input and output spaces of a logic function *f*, respectively. A logic function *f* maps x to ***y****,* expressed as (*y*_*1*_, *y*_*2*_, …, *y*_*n*_) = *f(x*_*1*_, *x*_*2*_, … *x*_*m*_*)*, where elements in ***x*** and ***y*** are binary variables, taking values from the set {0, 1}. The key concept behind GSPL-BEL is that valid states, which conform to the target logic function within the state space, i.e., *S*_*y*=*f(x)*_ = {(*x*_*1*_, *x*_*2*_, … *x*_*m*_, *y*_*1*_, *y*_*2*_, …, *y*_*n*_) | f(*x*_*1*_, *x*_*2*_, … *x*_*m*_) = (*y*_*1*_, *y*_*2*_, …, *y*_*n*_)} should configure the system to the ground state *E*_*min*_. In contrast, other invalid states *S*_*y≠f(x)*_ = {(*x*_*1*_, *x*_*2*_, … *x*_*m*_, *y*_*1*_, *y*_*2*_, …, *y*_*n*_) | f(*x*_*1*_, *x*_*2*_, … *x*_*m*_) ≠ (*y*_*1*_, *y*_*2*_, …, *y*_*n*_)} contribute a penalty energy *E*_*max*_ to the system, as follows:

1 $$\begin{array}{*{20}c} {E = \left\{ {\begin{array}{*{20}c} {E_{min} ,\quad S_{y = f\left( x \right)} } \\ { E_{max} ,\quad S_{y \ne f\left( x \right)} } \\ \end{array} } \right.} \\ \end{array}$$

It is important to note that *E*_*min*_ < *E*_*max*_. By fully harnessing the capabilities of many-body interactions, this model can achieve the utmost simplicity in the energy landscape for the target logic, as illustrated in Fig. 1e. In this energy landscape, all correct solutions to the computational problem are mapped to the ground state, while all wrong solutions are mapped to the excited energy state.

When a system designed from the GSPL-BEL model reaches thermal equilibrium at a finite temperature, the steady-state probability distribution of the system’s states can be characterized using the Boltzmann distribution:

2 $$\begin{array}{*{20}c} {P\left( {\left\{ s \right\}} \right) = \frac{{\exp \left( { - \frac{{E\left( {\left\{ s \right\}} \right)}}{T}} \right)}}{{\mathop \sum \nolimits_{i,j} \exp \left( { - \frac{{E\left( {\left\{ s \right\}} \right)}}{T}} \right)}}} \\ \end{array}$$

where *T* is a pseudo-temperature parameter that reflects the degree of stochasticity of the system in the context of GSPL. In the subsequent section, we will introduce how each Ising cell is implemented with a p-bit device.

Following this, by substituting the binarized energy levels, namely *E*_*min*_ and *E*_*max*_, into Eq. (2), we can derive the theoretical solutions for the statistical probabilities associated with the valid and invalid states, as follows:

3a $$\begin{array}{*{20}c} {P\left( {S_{y = f\left( x \right)} } \right) = \frac{{\exp \left( { - \frac{{E_{min} }}{T}} \right)}}{{N_{{S_{y = f\left( x \right)} }} \cdot \exp \left( { - \frac{{E_{min} }}{T}} \right) + N_{{S_{y \ne f\left( x \right)} }} \cdot \exp \left( { - \frac{{E_{max} }}{T}} \right)}}} \\ \end{array}$$

3b $$\begin{array}{*{20}c} {P\left( {S_{y \ne f\left( x \right)} } \right) = \frac{{\exp \left( { - \frac{{E_{max} }}{T}} \right)}}{{N_{{S_{y = f\left( x \right)} }} \cdot \exp \left( { - \frac{{E_{min} }}{T}} \right) + N_{{S_{y \ne f\left( x \right)} }} \cdot \exp \left( { - \frac{{E_{max} }}{T}} \right)}}} \\ \end{array}$$

where *N*_*Sy* = *f(x)*_ and *N*_*Sy ≠ f(x)*_ are the number of valid and invalid states in the state space, respectively.

To illustrate the procedure, a simple three-cell logic system is used as a paradigmatic example. The configuration of this three-body interacting model is fully characterized by a set of connectivity parameters, represented by the set {*h*_*A*_, *h*_*B*_, *h*_*C*_, *J*_*AB*_,* J*_*AC*_*, J*_*BC*_, *K*_*ABC*_}. For the purpose of demonstrating the process of deriving the configuration parameters that encode the simplest energy landscape, we consider an AND gate with input nodes *s*_*1*_ and *s*_*2*_, and an output node *s*_*3*_. The initial step in this process involves the summation of all candidate energies *E*_*s1s2s3*_ within the state space:

4 $$\begin{aligned} E_{{f\left( {AND} \right)}} & = \sum E_{{S_{{y = f\left( {AND} \right)}} }} + \sum E_{{S_{{y \ne \left[ {f\left( {AND} \right)} \right]}} }} \\ & = \begin{array}{*{20}c} {\left\{ {E_{{\min \left( {000} \right)}} + E_{{\min \left( {010} \right)}} + E_{{\min \left( {100} \right)}} + E_{{\min \left( {111} \right)}} } \right.} \\ {\left. { + E_{{\max \left( {001} \right)}} + E_{{\max \left( {011} \right)}} + E_{{\max \left( {101} \right)}} + E_{{\max \left( {110} \right)}} } \right\}} \\ \end{array} \\ \end{aligned}$$

The total energy of the system needs to be binarized to *E*_*min*_ and *E*_*max*_, after applying constraints. Specifically, when the state corresponds to one of the four valid configurations, namely (*s*_*1*_* s*_*2*_* s*_*3*_) = (0 0 0), (0 1 0), (1 0 0), and (1, 1, 1), the energy associated with that particular state configuration is assigned to the ground state energy *E*_*min*_. Conversely, for the remaining four undesirable states, the system is configured to exhibit an energy level of *E*_*max*_. To express the energy in terms of variables that satisfy these constraints, we employ Boolean ring conversion, in which the binary states 0 and 1 are represented as 1 − *s* and* s*, respectively:

5 $$\begin{aligned} E_{f\left( x \right) \leftrightarrow AAND} & = E_{min} \left[ {\left( {1 - s_{1} } \right)\left( {1 - s_{2} } \right)\left( {1 - s_{3} } \right) + \left( {1 - s_{1} } \right)\left( {s_{2} } \right)\left( {1 - s_{3} } \right)} \right. \\ & \quad \left. { + \left( {s_{1} } \right)\left( {1 - s_{2} } \right)\left( {1 - s_{3} } \right) + \left( {s_{1} } \right)\left( {s_{2} } \right)\left( {s_{3} } \right)} \right] \\ & \quad + E_{max} \left[ {\left( {1 - s_{1} } \right)\left( {1 - s_{2} } \right)\left( {s_{3} } \right) + \left( {1 - s_{1} } \right)\left( {s_{2} } \right)\left( {s_{3} } \right)} \right. \\ & \quad \left. { + \left( {s_{1} } \right)\left( {1 - s_{2} } \right)\left( {s_{3} } \right) + \left( {s_{1} } \right)\left( {s_{2} } \right)\left( {1 - s_{3} } \right)} \right] \\ \begin{array}{*{20}c} \begin{aligned} & = \left( {E_{max} - E_{min} } \right)s_{3} + \left( {E_{max} - E_{min} } \right)s_{1} s_{2} \\ & \quad - 2\left( {E_{max} - E_{min} } \right)s_{1} s_{2} s_{3} + E_{min} \\ \end{aligned} \\ \end{array} \\ \end{aligned}$$

To ensure compatibility with the Ising model’s conventions, which employ a bipolar representation format for variables, it is necessary to convert the energy function from the binary format to bipolar format using the transformation *s* = (*m* + 1)/2:

6 $${E}_{AND}=\frac{1}{4}{(E}_{max}-{E}_{min})\left[{m}_{3}-{m}_{1}{m}_{3}-{m}_{2}{m}_{3}-{{m}_{1}{m}_{2}m}_{3}+2\frac{{E}_{max}+{E}_{min}}{{E}_{max}-{E}_{min}}\right]$$

where *m* represents the bipolar value − 1 and + 1. Concurrently, the energy of a many-body-interacting Ising system is defined as:

7 $$\begin{aligned} E\left( {\left\{ s \right\}} \right) & = - \left( {\mathop \sum \limits_{i} h_{i} m_{i} + \mathop \sum \limits_{i < j} J_{ij} m_{i} m_{j} + \mathop \sum \limits_{i < j < k} K_{ijk} m_{i} m_{j} m_{k} } \right. \\ & \quad \left. { + \mathop \sum \limits_{i < j < k < l} L_{ijkl} m_{i} m_{j} m_{k} m_{l} + \cdots } \right) \\ \end{aligned}$$

The energy of this 3-node AND gate is:

8 $$\begin{aligned} E_{AND} & = - \left( {h_{1} m_{1} + h_{2} m_{2} + h_{3} m_{3} } \right) - \left( {J_{12} m_{1} m_{2} + J_{13} m_{1} m_{3} + J_{23} m_{2} m_{3} } \right) \\ & \quad - K_{123} m_{1} m_{2} m_{3} \\ \end{aligned}$$

The configuration parameters of the system can be determined by calculating the ratio of coefficients for the bias [*h*], pairwise [*J*], and three-body term *K*_*123*_. The constant term in the calculation exactly corresponds to the ground state of the system. This direct mapping approach exhibits a high degree of generalizability and can be readily extended to any target logic function, provided that its truth table is known a priori. We have packaged this framework that can determine the configuration parameters of the Ising model based on the target logic function into an executable program for interested readers to validate and utilize^39^.

### Examples of GSPL-BEL using graphic representations

We have introduced a comprehensive framework for determining the connectivity of GSPL circuits by designing energy functions. To demonstrate the operation of the GSPL-BEL model, we will investigate small-scale GSPL gates with the number of nodes ranging from 3 to 5 and provide their corresponding graphical representations. Moreover, a GSPL library has been established, which serves as a valuable resource that can be directly utilized for further studies in this field.

The majority gate serves as a core component in a wide range of applications, including image processing^40^ and brain-inspired computing^41^. We explore a potential implementation of a majority gate based on the GSPL-BEL model. After normalizing the connectivity strength by setting *E*_*min*_ and *E*_*max*_ in Eq. (1) to 0 and 1, respectively, the energy function of a 3-input majority gate, composed of nodes *A*, *B*, *C,* and *O*, can be described as follows:

9 $$\begin{array}{*{20}c} {E_{Majority} = - m_{A} m_{O} - m_{B} m_{O} - m_{C} m_{O} + m_{A} m_{B} m_{C} m_{O} - 2.} \\ \end{array}$$

**Figure 2:**
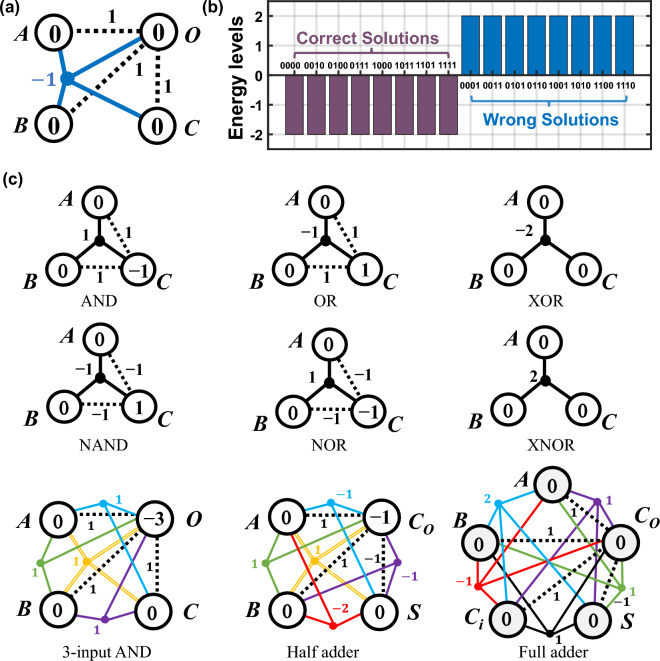
(**a**) Graphic representation and (**b**) energy landscape of a 3-input and 1-output majority gate designed from the GSPL-BEL model. The sign of integers marked on the connection lines indicates the polarity of interactions received from other nodes, which can be negative or positive. (**c**) Configuration library of many-body-interacting fundamental GSPL-BEL gates.

The absolute values of the coefficients in the first three terms and the fourth term define the interaction strength of pairwise and four-body interactions, respectively. The overall configurations of the system can be succinctly captured by the graphical representation shown in Fig. 2a. This model is composed of four nodes, and the sign of interaction strength represents the direction of the interaction received from neighboring cells. For instance, cell *A* receives a negative four-body-interaction signal from nodes *B*, *C,* and *O*.

The energy, *E*_*ABCO*_, for each state configuration in the state space of this 4-node majority gate can be computed using Eq. (7). As illustrated in Fig. 2b, there are two distinct energy levels, in which all valid and invalid states are degenerate. The valid states configure the system to the ground state energy *E*_*min*_ =  − 2, which is in agreement with the constant term in Eq. (9). In contrast, the invalid states are collectively mapped to the maximum energy level, *E*_*max*_, which takes the value of + 2. As dictated by the principles of statistical mechanics, the excited states corresponding to higher energy levels exhibit a lower probability of being occupied at thermal equilibrium. Consequently, we can anticipate the emergence of a binarized probability landscape.

To further demonstrate the versatility and potential of the GSPL-BEL model, additional examples of logic families, ranging from 3-node to 5-node configurations, are provided in Fig. 2c. By customizing the configuration parameters based on specific truth tables, we first design various logic functions within three-body-interacting systems, including AND, NAND, OR, NOR, XOR, and XNOR. Upon introducing an additional node, more complex operations can be realized, such as the majority function discussed earlier, 3-input and 1-output AND operation, and half addition operation, by incorporating four-body interactions into the 4-node network. Furthermore, the half addition operation can be further upgraded to a full addition operation by introducing an additional node to serve as the carry-in node *Ci*. This 5-body-interacting Full adder can propagate any carry generated from lower-order bits to higher-order bits, making it suitable for multi-digit binary addition. Under the proposed GSPL-BEL model. These small-scale many-body-based logic components consistently exhibit a binarized energy landscape and could serve as building blocks of combinatorial GSPL circuits. For example, by combing the many-body-based AND gates, Half adders, and Full adders, integer factorizers can be constructed to efficiently solve the integer factorization problem. Besides, the NOT gate, three-body-based AND gate, and three-body-based OR gate can be logically synthesized to create solvers for Boolean satisfiability problems. The simplification effects of the many-body-based design on the energy landscape of logically synthesized factorizers have been investigated in our previous research^19^. However, it is imperative to emphasize that the development of probabilistic models for larger-size logic circuits in this work does not involve a logic synthesis process, as the combination of basic gates would introduce additional and unnecessary energy levels, thereby significantly complicating the energy landscape. Moreover, the solution space of the factorization problem would expand due to the involvement of auxiliary nodes. Our primary objective is to present a design solution that achieves the simplest energy landscape for GSPL circuits of any size with minimal overhead of nodes, and to validate the functionality of GSPL-BEL using p-bit devices.

## Hardware implementations

### Generic p-bit device

**Figure 3:**
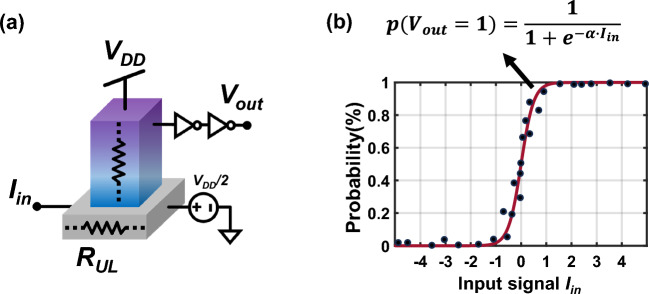
P-bit device and its electrical response behavior. (**a**) Generic model of the p-bit building block for the construction of GSPL circuits. (**b**) Sigmoidal response of the p-bit device with respect to the input signal, where α is a parameter reflecting the tilt degree of the *S*-shape curve and it is determined by the stochasticity of the specific p-bit devices.

In this section, the processes of translating the graphical representations of GSPL circuits into electronic elements will be described in detail. Figure 3a depicts a generic three-terminal p-bit device with an analog input terminal and a digital output terminal, which serves as the fundamental building block of GSPL circuits. As illustrated, the output signal from this device is a binary voltage, accepting only two distinct values: 1 and 0, representing the high voltage level *V*_*DD*_ and the low voltage level 0, respectively. This device is capable of constantly generating fluctuating bitstreams composed of 1s and 0s. By adjusting the direction and strength of the input signal, the probability of producing 1s can be tunable. This stochastic behavior adheres to a sigmoidal relation and can be described as follows^6^:

10 $$\begin{array}{*{20}c} {s_{i} \left( t \right) = \left. {sgn\left\{ {rand\left( { - 1, 1} \right)} \right\} + tanh\left[ {I_{in} \left( t \right)} \right]} \right\}} \\ \end{array}$$

where *I*_*in*_ represents a current signal, and rand (− 1, + 1) denotes a uniformly distributed random number between − 1 and + 1.

The probability of obtaining an output of 1 in response to the input signal is determined statistically by averaging the output values over an extended period of time. To date, a variety of p-bit devices have demonstrated their functionality and applicability in the field of probabilistic computing. These devices encompass thermal noise-driven stochastic magnetic tunnel junctions^5,7–9,42,43^, programmed microcontrollers^44^, CMOS-based^20,32^ FPGA-based^45^, and other emerging probabilistic devices^46–51^. Networks and systems constructed upon these devices have exhibited remarkable effectiveness in solving a diverse range of hard computational problems, including integer factorization^6,17,19,21^, combinatorial optimization problems^18,52,53^, Bayesian inference^8,54–58^, and machine learning^3,59,60^. However, to minimize the time required to converge to solutions, the fluctuation time between 0 and 1 s of p-bit devices should be as short as possible, while still adhering to the constraints imposed by the circuit design^61^. In this work, we start by characterizing the probabilistic characteristics of a given p-bit device through a fitted sigmoidal curve depicted in red, or a Lookup table that reflects the discrete data points, shown in black, as illustrated in Fig. 3b. Subsequently, we develop a Verilog-A behavioral model in Cadence Virtuoso after modeling and packaging the device to a modular cell. Finally, the cells are assembled into probabilistic circuits with various computational functions, based on a pre-designed network structure that incorporates many-body interactions. Utilizing probability statistics grounded in Boltzmann’s law, we evaluate and analyze the overall performance of the GSPL-BEL at the circuit level. The implementation of many-body effects based on CMOS electronic elements will be discussed in the next section.

### Many-body interactions

Building upon the utilization of p-bit devices to implement Ising cells, another critical step in designing many-body interacting GSPL circuits lies in determining suitable hardware implementations for many-body interactions among cells. To streamline the design of network connections, we opt for a p-bit device with an analog current input and digital voltage output to function as the Ising cell. This kind of device can simplify the hardware implementation of interactions within two dimensions, as the conversion from the voltage output of the cell to the current input of neighboring interacting cells can be easily implemented through a passive resistor network^6^. In this configuration, the feedback current serves as the carrier of the cell-to-cell interaction. During operation, cells are updated sequentially, and the strength of incentive for cell *m*_*i*_ is determined by the cumulative interaction values of its neighboring cells:

11 $$\begin{array}{*{20}c} {I_{i} \left( t \right) = h_{i} + \mathop \sum \limits_{j} J_{ij} s_{j} \left( t \right) + \mathop \sum \limits_{j, k} C_{ijk} s_{j} \left( t \right)s_{k} \left( t \right) + \mathop \sum \limits_{j, k,l} D_{ijkl} s_{j} \left( t \right)s_{k} \left( t \right)s_{l} \left( t \right) + \cdots } \\ \end{array}$$

**Figure 4:**
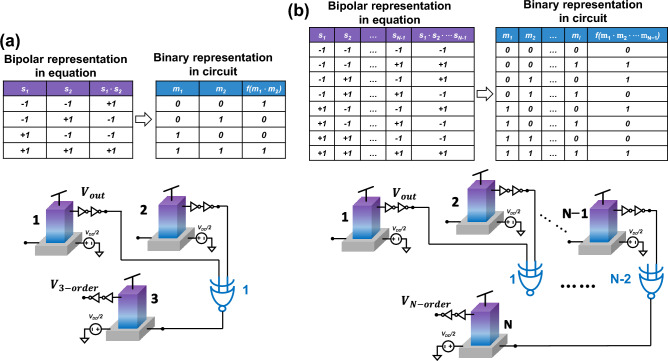
Hardware implementation of many-body interactions. (**a**) The differences between mathematic and circuit representations of the three-body effect. One XNOR gate in blue color is used to implement the three-body interaction. (**b**) *N*-2 serially connected XNOR gates are used to implement the *N*-body interaction.

Focusing specifically on the third term representing the three-body interaction and an *N*-body interaction, the contribution of one of their respective branches to the total accumulated current is expressed as follows:

12a $$\begin{array}{*{20}c} {I_{i, 3 - body} = C_{123} s_{1} s_{2} } \\ \end{array}$$

12b $$\begin{array}{*{20}c} {I_{{i,N{ - }body}} = Z_{{N{ - }body}} \cdot \mathop \prod \limits_{1}^{N - 1} s_{i} } \\ \end{array}$$

where *Z*_*N-body*_ represents the coefficient for the *N*-body interaction. In the top left of Fig. 4a, the theoretical values of nodes *m*_*1*_, *m*_*2*_, and *m*_*1*_·*m*_*2*_ after the three-body interaction are represented in the bipolar format. However, to facilitate the practical implementation of the circuit, these values must undergo a conversion to the binary format using the function *f(s*_*1*_*,s*_*2*_*)*, as the output information of cells is encoded using digital 0 and 1 in the circuit. Interestingly, this function perfectly matches the operation of a conventional XNOR gate, as illustrated in the bottom left of Fig. 4a. Furthermore, by cascading *N − *2 XNOR gates in series, the hardware implementation of many-body interactions can be scaled up to accommodate an *N*-body system. Figure 4b provides a visual representation of this implementation, wherein the output signals of cells *s*_*1*_ and *s*_*2*_ are first processed by the first XNOR gate and subsequently fed to subsequent XNOR gates. The final output signal following the *N*-body interaction is obtained from the output of the last XNOR gate, namely the (*N*-2)*th* XNOR gate.

## Results and discussion

### Example of the GSPL-BEL with electronic elements

**Figure 5:**
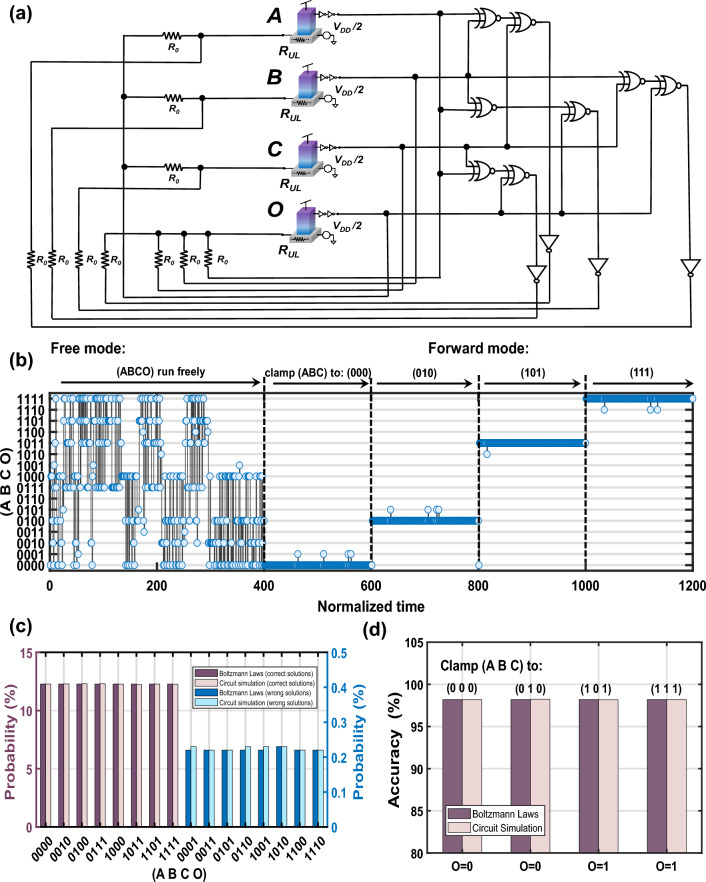
(**a**) Schematic of the GSPL-BEL-based majority gate, in which the interactions are implemented in hardware with resistors together with conventional XNOR gates. (**b**) Real-time waveform clip of the majority gate operating in the free and forward modes. (**c**) Statistical probability distributions of the majority gate operating in the free mode (**d**) and four stages of the forward mode. All statistics results are obtained by averaging 10^7^ sampling points in the time domain. The clamping operation is enabled by injecting a strongly positive or negative current to corresponding p-bits.

Figure 5a presents the schematic of a 4-node Majority gate designed based on the proposed GSPL-BEL model, wherein all graphical representation information has been translated into electronic components. Specifically, we have combined a resistor network with XNOR gates to implement the four-body interactions. In the circuit implementation, we have made several simplifying assumptions to facilitate the analysis and simulation of the system: (1) The resistance of the underlying layer, labeled as *R*_*UL*_, is considered negligible. Therefore, the voltage of the input terminal for each cell can be fixed to *V*_*DD*_/2, which is nearly equal to the bias voltage applied at the third terminal. When the input current equals 0 (*V*_*in*_ = *V*_*DD*_/2), a 50% probability of obtaining a logical 1 can be achieved. (2) The response time, which includes the total time for cell retention and fluctuation, is assumed to be approximately 1 ns^12,13^. (3) We also assume that the transmission time of XNOR gates used to implement many-body interactions is significantly shorter than the response time of cells and that there is no delay across CMOS gates. Circuit simulations are conducted using the HSPICE simulator in Cadence Virtuoso, with detailed parameters summarized in Table 1.

**Table 1:** Parameters used in circuit simulations.

Electronic elements	Parameters	Value
P-bit building block	Tilt degree parameter *α*	4 × 10^6^
	Fluctuation time (ns)	1
Resistor	*R*_*0*_ (Ω)	1 × 10^6^
Voltage source	Global voltage (*V*_*DD*_)	1

For this majority gate, we explored two operating modes: the free mode and the forward mode. In the free mode, none of the nodes (*A*, *B*, *C*, *O*) are clamped, allowing them to operate freely. Conversely, by clamping the input nodes (*A*, *B*, *C*) to the four distinct configurations of interest, namely (0, 0, 0), (0, 1, 0), (1, 0, 1), and (1, 1, 1), we can examine the probabilistic gate’s response and evaluate its accuracy in producing the expected output at these different stages. As illustrated in Fig. 5b, when running in the free mode, the majority gate continuously explores all 2^4^ = 16 possible state configurations within the state space, with a higher emphasis on the 8 candidate configurations that conform to the truth table. As demonstrated by the circuit simulation results in Fig. 5c, the average statistical probabilities of the 8 valid state configurations and the 8 invalid state configurations are 12.28% and 0.22%, respectively, which perfectly match the theoretical values of 12.28% and 0.22% calculated using Eq. (3). The emergence of this probability binarization is attributed to our proposed GSPL-BEL model’s ability to map the energies of valid and invalid states to two distinct energy levels. Furthermore, as shown in Fig. 5d, when the majority gate operates in the forward mode, the probabilities of output 0, 0, 1, and 1 for the four stages under investigation are 98.20%, 98.23%, 98.17%, and 98.19%, respectively. These values exhibit a strong agreement with the theoretical probability of 98.19%. The close correspondence between the above probabilities obtained by circuit simulation and the theoretical values is a testament to the robustness and predictability of the GSPL-BEL model.

### Non-logic synthesis GSPL-BEL

Apart from the free mode and forward running mode, the most remarkable feature of probabilistic circuits designed using the GSPL-BEL framework is their ability to operate in the reverse mode. This mode of operation enables the circuit to effectively infer the most likely inputs or parameters that give rise to the observed outputs by propagating information backward through the probabilistic network. This capability provides a powerful tool for inference, optimization, and decision-making, in contrast to traditional logic gates, which are limited to unidirectional forward operation. Furthermore, the GSPL-BEL model also allows for circumventing the logic decomposition and logic synthesis steps typically required in conventional logic circuit design.

**Figure 6:**
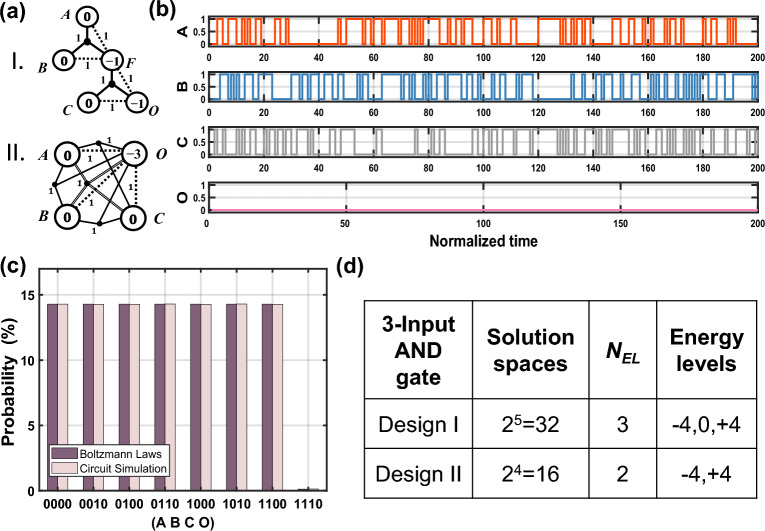
(**a**) Logic schematics of 3-input and 1-output AND gates involving multi-body effects. Design I is built by connecting two 2-input and 1-output AND gates serially, whereas design II is directly implemented based on the GSPL-BEL model. (**b**) Real-time waveform clip and (**c**) statistical probability distributions of the design II-based 3-input AND when the output O is clamped to 0. (**d**) Comparison of key energy metrics. All statistics results are obtained by averaging 10^7^ sampling points in the time domain.

To more intuitively illustrate these advantages of the GSPL-BEL model, we consider a 3-input and 1-output AND gate involving many-body effects. As shown in Fig. 6a, there are two design schemes: design I is realized by logically synthesizing two two-input AND gates, requiring a total of 5 nodes and involving up to 3-body interactions; while design II is directly implemented based on the GSPL-BEL model, requiring only 4 nodes but involving up to 4-body interactions. Figure 6b shows the output waveforms of the three input nodes *A*, *B*, and *C* of design II when the output *O* is clamped to 0. After averaging over the time domain, we can obtain the probability distribution corresponding to different state configurations, as shown in Fig. 6c. It can be observed from the figure that the system nearly uniformly explores the 7 state configurations that satisfy *O* = 0. Simulation results indicate that the average probability of these 7 candidate state configurations is 14.28%, while the probability of the wrong solution (1110) is only 0.005%, which is in complete agreement with the theoretical calculations. A detailed comparison of these two designs in terms of solution spaces and number of energy levels (*N*_*EL*_) can be seen in Fig. 6d.

Further, to comprehensively evaluate the functionality of the GSPL-BEL model in a broader range of logic circuits, we investigate its application in multipliers. A notable feature of GSPL-BEL-based multipliers is their ability to operate in the reverse mode, enabling them to function as invertible multipliers or integer factorizers. This unique capability holds promising potential in various domains, such as encryption and machine learning.

**Figure 7:**
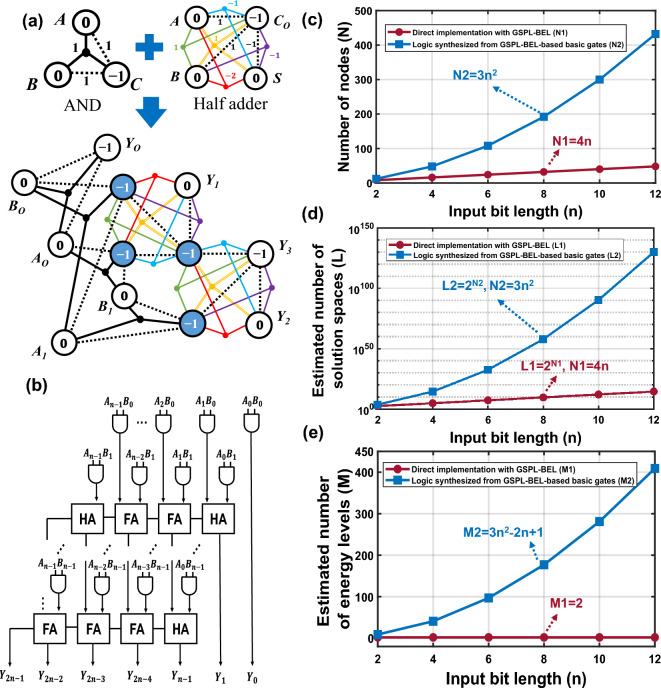
(**a**) A 2-bit × 2-bit invertible multiplier logically synthesized from 4 AND gates and 2 Half adders. (**b**) Schematic of a *n*-bit × *n*-bit adder-based invertible multiplier/integer factorizer. (**c**) Comparison of number of nodes. (**d**) Comparison of estimated solution spaces. (**e**) Comparison of the estimated number of energy levels.

As an initial step, we construct a 2-bit × 2-bit invertible multiplier using the logic synthesis method, which requires a total of 12 nodes. The interconnection of basic logic gates is achieved by merging the common nodes, as illustrated in Fig. 7a. The auxiliary nodes, depicted in blue, serve as bridges to facilitate the connection between different gates. A more general *n*-bit × *n*-bit multiplier can be developed based on the logic schematic presented in Fig. 7b, which outlines the key components and architecture of the multiplier. This architecture comprises *n*^2^ AND gates, *n* Half adders, and *n*(*n* − 2) Full adders, resulting in a total consumption of 3n^2^ nodes. In contrast, the GSPL-BEL model offers an alternative approach that bypasses the conventional framework of constructing combinational logic circuits through logic synthesis, which can be cumbersome and time-consuming. Consequently, the GSPL-BEL model provides the most compact design for the multiplier, minimizing overhead by determining the number of nodes solely based on the sum of input and output terminals. Unlike the polynomial growth observed in the conventional logic synthesis method, the GSPL-BEL-based model exhibits a linear node expansion of *N*_*1*_ = 4*n* relative to the multiplier’s size, as depicted in Fig. 7c. A significant advantage stemming from the reduction in required nodes is the substantial contraction of the solution space. For example, in the case of a 2-bit × 2-bit invertible multiplier operating in a free mode, the solution space can be reduced from 4096 to 256. The general relationship between the input bit length and the estimated number of solution spaces is presented in Fig. 7d.

Moreover, when directly implementing the multiplier using the GSPL-BEL model, the number of energy levels can be maintained at 2, even as the size of the multiplier grows linearly from 2-bit, as illustrated in Fig. 7e. This is attributed to the corresponding increase in the dimensionality of available many-body effects as the number of nodes increases with the growth of the multiplier’s size. As a result, for multipliers of any size, the energy can be effectively mapped to only two discrete energy levels, yielding the simplest energy landscape. Finally, we compare the factorization accuracy of a 2-bit × 2-bit multiplier with the output clamped to 6 in the reverse mode. Among the three implementations, the non-logic synthesis method provides the highest accuracy for the solutions (*A*, *B*) = (2, 3) and (3, 2) =  ~ 50% calculated by Eq. (3), whereas the accuracy for the logic synthesis approach based on two-body-based basic gates is 37.80%.

**Figure 8:**
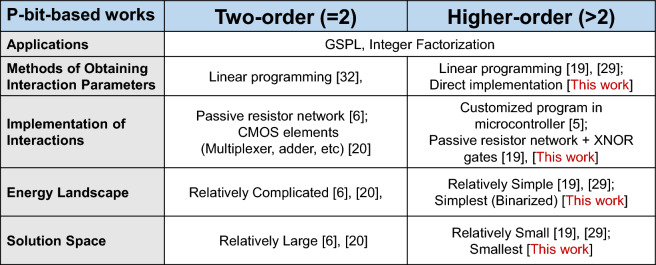
Comparison of p-bit-related works in the area of GSPL.

From the above circuit simulation results and theoretical derivation results, it can be seen that compared with the logic synthesis method based on many-body basic logic gates, the GSPL-BEL model has significant advantages in terms of the number of nodes, solution space, energy landscape simplicity, and computation accuracy. In fact, these advantages become more prominent when compared to combinational circuits based on two-body-interaction basic logic gates, as summarized in Fig. 8. However, we also note that as the scale of GSPL increases, the dimensionality of many-body effects in the GSPL-BEL model also increases accordingly. This may lead to more complicated hardware implementation of higher-order interactions. To ensure the efficient implementation of the GSPL-BEL model in large-scale GSPL circuits, future work can explore new circuit design techniques and hardware architectures, such as updating clocks for many-body effects^31^, to address the challenges of implementing many-body interactions while maintaining the advantages of the GSPL-BEL model in terms of number of nodes, solution space size, and energy landscape simplicity. Despite facing this challenge, the tremendous potential and unique advantages demonstrated by the GSPL-BEL model in the field of probabilistic computing still make it a highly attractive research direction worthy of further in-depth exploration and development.

## Conclusion

In this study, we introduce the GSPL-BEL model, a novel approach that incorporates many-body interactions, drawing from the foundational principles of ground-state computation and energy minimization. This model extends the interaction dimensionality within the Ising model-based p-bit network, transitioning from traditional two-body interactions to more advanced many-body interactions. This expansion provides greater flexibility and freedom in describing the energy function of the target GSPL. We demonstrate a robust implementation of the many-body interactions through a simple cascade arrangement of conventional XNOR gates, ensuring practical feasibility. Theoretical calculations based on Boltzmann’s law and rigorous statistical circuit simulations validate the GSPL-BEL model’s efficiency across various functions and system sizes. Notably, the model provides a compact design solution for probabilistic circuits by bypassing conventional logic synthesis methods, which significantly reduces the solution space size, leading to more efficient computation. Moreover, by fully harnessing the potential of many-body interactions, the GSPL-BEL model achieves binary simplification of energy landscapes for arbitrary logic. Looking ahead, our design showcases promise as a fault-tolerant, multifunctional, and efficient computational model for probabilistic applications.

### Supplementary Information

Supplementary Information.

## Data Availability

The data that support the results of this study are available from the corresponding author upon reasonable request.
