# Supplementary material for: Direct design of ground-state probabilistic logic using many-body interactions for probabilistic computing
Source: Sci Rep. 2024 Jul 2;14:15076. doi: 10.1038/s41598-024-65676-z (PMC11219996; doi:10.1038/s41598-024-65676-z)
Supplement: Supplementary file 1 — Supplementary Information. [file 41598_2024_65676_MOESM1_ESM.docx]

Supplementary Materials

Direct Design of Ground-State Probabilistic Logic Using Many-Body Interactions for Probabilistic Computing

Yihan He, Sheng Luo, Chao Fang, Gengchiau Liang*

*Corresponding Author: [gcliang@nycu.edu.tw](mailto:gcliang@nycu.edu.tw)

This supplement includes:

1. Circuit simulation results of the 2-input AND gate designed using the GSPL-BEL model in the free mode, reverse mode, and forward mode.
2. Circuit simulation results of the 3-input AND gate designed using the GSPL-BEL model in the free mode, reverse mode, and forward mode.
3. Circuit simulation results of the 3-input Majority gate designed using the GSPL-BEL model in the free mode, reverse mode, and forward mode.
4. 2-input AND gate

Fig. S1 Circuit simulation results of the 2-input AND gate designed using the GSPL-BEL model. (a) The gate operates in free mode, (b) reverse mode with C clamped to 0, (c) reverse mode with C clamped to 1, and (d) forward mode with (A B) clamped to (0 1). The left, middle, and right panels are respectively the real-time waveform fragments of A, B, and C, the real-time exploration fluctuations of (A B C), and the statistical probability distribution of all candidate solutions. All statistics results are obtained by averaging 10^6^ sampling points in the time domain.

1. 3-input AND gate

Fig. S2 Circuit simulation results of the 3-input AND gate designed using the GSPL-BEL model. (a) The gate operates in free mode, (b) reverse mode with O clamped to 0, and (c) forward mode with (A B C) clamped to (0 1 1). The top left, bottom left and right panels are respectively the real-time waveform fragments of A, B, C, and O, the real-time exploration fluctuations of (A B C O), and the statistical probability distribution of all candidate solutions. All statistics results are obtained by averaging 10^6^ sampling points in the time domain.

1. 3-input Majority gate

Fig. S3 Circuit simulation results of the 3-input Majority gate designed using the GSPL-BEL model. (a) The gate operates in free mode, (b) reverse mode with O clamped to 0, and (c) forward mode with (A B C) clamped to (1 0 1). The top left, bottom left and right panels are respectively the real-time waveform fragments of A, B, C, and O, the real-time exploration fluctuations of (A B C O), and the statistical probability distribution of all candidate solutions. All statistics results are obtained by averaging 10^6^ sampling points in the time domain.
